# Hybrid Process of Adsorption/Coagulation/Ceramic MF for Removing Pesticides in Drinking Water Treatment—Inline vs. Contact Tank PAC Dosing

**DOI:** 10.3390/membranes11020072

**Published:** 2021-01-20

**Authors:** Rui M. C. Viegas, Margarida Campinas, Rosário Coelho, Helena Lucas, Maria João Rosa

**Affiliations:** 1 Water Quality and Treatment Laboratory, Urban Water Unit, Hydraulics and Environment Department, LNEC—National Civil Engineering Laboratory, 1700-066 Lisbon, Portugal; rviegas@lnec.pt (R.M.C.V.); mjrosa@lnec.pt (M.J.R.); 2 AdA—Águas do Algarve S.A., Rua do Repouso, 8000-302 Faro, Portugal; r.coelho@adp.pt (R.C.); h.lucas@adp.pt (H.L.)

**Keywords:** powdered activated carbon/coagulation/microfiltration, hybrid membrane process, pesticides, ceramic membranes, inline, tank, PAC dosing options

## Abstract

Two pilot trials of powdered activated carbon (PAC)/(coagulation)/ceramic microfiltration were conducted to compare continuous 10–12 mg/L PAC inline dosing with 8–10 mg/L dosing to a 2 h-contact tank. Two low turbidity/low natural organic matter (NOM, total organic carbon <2 mg C/L) surface waters spiked with 7.2–10.3 µg/L total-pesticides were tested and the dosing options were compared towards operational performance, average removal of pesticides and NOM and costs. Removal differences between the two PAC dosing options depended on pesticides’ amenability to adsorption and NOM characteristics (254 nm absorbance, A254). Waters containing low A254-absorbing NOM and only pesticides amenable to adsorption showed very high removals (all pesticides ≥93%) and no significant differences between the two PAC dosing options. Waters containing higher A254-absorbing NOM and high loads of pesticides less amenable to adsorption (dimethoate, bentazone) required higher inline PAC dose. Those or more severe conditions may require PAC doses higher than tested to comply with the Drinking Water Directive limits for pesticides. Cost analysis showed PAC inline dosing is more cost-effective than PAC dosing to the contact tank when identical PAC dose is sufficient or when the doses are low, even if 50% higher for inline dosing, and the plant is small.

## 1. Introduction

Agriculture is a major source of pesticides in European surface and groundwaters, important drinking water resources, but there is still limited information available on pesticide contamination and a lack of reliable and comparable data [1]. A recent study on global variations in pesticide regulations concluded that about 34% of the world population in about 56% of the world’s nations are not adequately protected from the human health risks of pesticide-contaminated drinking water [2]. In agreement, a recent 5-years monitoring study in The Netherlands, covering 408 pesticides and 52 metabolites in groundwater and surface water used as drinking water sources, detected pesticides and/or metabolites in two thirds of the abstraction areas, with one third of the areas exceeding 0.1 µg/L, the Water Framework Directive water quality standards [3]. Moreover, a review of pesticides’ monitoring studies of surface waters worldwide [4] showed a critical occurrence of atrazine and its metabolites metalochlor, chlorpyrifos and tebuconazole, and high concentrations and frequency of diuron (0.03–22,770 ng/L) and of the insecticide dimethoate (0.57–61,200 ng/L). It is known that pesticides can lead to harmful effects in aquatic ecosystems and risks to human health, as they are persistent, bioaccumulative, mobile in the environment, are potential endocrine-disruptors and may cause severe health problems, such as cancer, infertility, impairment of memory and nervous system, amongst others [1,4,5]. In addition, health concerns have recently arisen on mixtures of low concentrations of pesticides as they may result in complex substances more toxic than each single compound [4,5,6]. Although little is currently known about the safety of low-dose pesticides’ mixtures, some recent results have shown prolonged exposure to pesticide mixtures may cause adverse neurobehavioral effects, even at permitted levels [5]. Besides the above, climate change is expected to affect pesticides’ patterns in waters as rain intensity and frequency may interfere and or aid in pesticides’ transport and mobility [4]. Therefore, finding suitable and cost-effective water treatment, with low vulnerability and high adaptation capacity, is crucial in the current climate uncertainty context of increasing limited water resources and challenging water quality requirements.

Powdered activated carbon (PAC) based solutions are amongst the best available technologies for controlling organic microcontaminants in conventional water treatment plants (WTPs) due to PAC high adsorption ability for a wide range of microcontaminants [7,8,9,10], high flexibility and easy implementation, simultaneously avoiding the potential formation of undesired by-products with unknown toxicity (sometimes more toxic than the parent compounds). A reliable downstream filtration is nevertheless required to retain PAC fines, particularly in challenging conditions for coagulation to occur, such as low turbidity/low organic matter/low alkalinity waters [11]. The hybrid PAC/coagulation/ceramic microfiltration (MF) process is a very appealing barrier for upgrading conventional water treatment plants. On one hand, it enhances organic microcontaminants’ removal by PAC and, on the other hand, it ensures higher and more reliable disinfection capacity (including for viruses and protozoa (oo)cysts) while enabling the use of smaller PAC particles, allowing faster adsorption kinetics and thus a better performance, with very efficient separation (via coagulation/microfiltration), even for PAC fines [10,12,13,14]. PAC/coagulation/ceramic MF has also showed stable operation and high adaptation capacity to water quality changes [15,16,17,18,19,20,21]. Although PAC/MF studies have mainly focused on conventional polymeric membranes, ceramic membranes are potentially more interesting for PAC long-term use due to their higher resistance to deterioration by biofilm growth and to surface abrasion by coarse particles circulation [17,22,23].

The existence of a PAC contact tank is seen as a requirement for increasing the contact time and achieve a maximum removal of contaminants with a PAC load as low as possible. Nevertheless, inline PAC dosing is an easier to implement solution that would allow cost savings and a smaller treatment footprint, which might be important for small plants or densely populated areas where space is a limiting factor. Cost functions earlier developed for PAC/(coagulation)/MF [20], with 10 mg/L PAC dosing to a contact tank (2 h contact time), yielded a total (investment and operation) cost of 0.07–0.11 €/m^3^ for 100,000 m^3^/d. However, investment costs, that could have a significant share, would be saved if inline PAC dosing could provide a significant microcontaminants’ removal. Although several authors have approached PAC dosing modes, the focus has been on single versus step or continuous PAC dosing, and the advantages/limitations of inline PAC dosing have not yet been sufficiently studied or contradicting results were reported. For instance, our previous work [24] with pilot-scale PAC/coagulation/ceramic MF for removing four pharmaceutical compounds from a secondary effluent showed +15% to +18% added removal with a PAC contact tank compared to inline PAC dosing. In turn, pilot-scale PAC/ultrafiltration studies carried out by Ivancev-Tumbas et al. [25] for removing p-nitrophenol from tap water revealed no significant differences between these two options when a continuous PAC dosing was used. Furthermore, Ellerie et al. [26] lab-scale dead-end MF studies with PAC pre-coated membranes showed a greater initial atrazine removal compared to adding PAC to a stirred tank, which could indicate better microcontaminants’ removals with inline PAC dosing than anticipated. More studies are necessary as different results may occur due to differences in background water matrix, microcontaminants’ characteristics, membrane systems and PAC characteristics and doses. Microcontaminants’ affinity for adsorption seems to depend not on a solute single parameter but on several properties determining the microcontaminants-PAC-NOM interactions, amongst them, hydrophobicity, charge, size, aromaticity and polarity [8,11,27,28,29]. Overall, in natural waters, PAC seems to be more efficient for the adsorption of neutral hydrophobic or positively charged compounds [11,28,29], the latter apparently through microcontaminants-PAC-NOM electrostatic interactions [28,29,30]. For low-hydrophobicity compounds, positively charged functional groups and low surface polar area and/or high number of aromatic rings seem to act as adsorption enhancers [11,27]. The influence of PAC contact time on microcontaminants’ removal was shown to depend on microcontaminants’ characteristics and NOM-microcontaminants competition, some compounds benefiting with contact time increase, others not being affected [29]. As NOM competition impacts are stronger for weakly adsorbing microcontaminants [31,32], PAC contact time is expected to be more important for those compounds. Testing different microcontaminants and waters is therefore extremely important for effectively comparing PAC dosing efficiency.

This paper aims at comparing two PAC dosing modes during a pilot PAC/coagulation/ceramic MF study, in terms of operational performance, average removal of pesticides and organic matter, and costs. “PAC dosing modes” (or options) refer to inline versus tank dosing, both continuous modes but providing very different PAC contact time, and not to single versus step or continuous PAC dosing commonly analysed in literature. PAC continuous dosing into a contact tank vs. PAC continuous inline direct addition were compared during two short-term trials conducted with two surface waters of low turbidity/low NOM spiked with a mixture of pesticides of different amenabilities to PAC adsorption. This study is believed relevant and with novelty to the current state of the art since: (i) it approaches a pressurized PAC/MF process with ceramic membranes, less studied for hybrid adsorption/membrane processes; (ii) it is a pilot study with natural waters (close to real world conditions), while many studies were conducted at lab scale and/or with synthetic waters; (iii) it compares inline versus tank PAC dosing in different water matrix conditions and targeting the removal of pesticides with different amenabilities to PAC adsorption.

## 2. Materials and Methods

### 2.1. Pesticides

European Union Member States monitor a considerable number of pesticides and metabolites in drinking water, which are selected at national level and are thus Member State specific. Nine pesticides, selected to be monitored in Portugal by the Portuguese Environment Agency, were studied. They were spiked into two surface waters feeding PAC/coagulation/ceramic MF pilot (Table 1).

According with our previous results with an identical pool of pesticides, though with other PAC in PAC/CFS application [11], different amenabilities to PAC adsorption are expected from those nine pesticides. The percentile distribution of removal efficiencies was therein represented and dimethoate and bentazone were amongst the compounds presenting less amenability to adsorption (P < 33), while chlortoluron, diuron, linuron and tebuconazole were amongst those more amenable to adsorption compounds (>P67). Similar conclusions were drawn from other PAC/(Alum)/MF trials with the PAC herein used [32].

Concentrated stock solutions (5 mg/L in deionized water) of the compounds (Sigma-Aldrich) were prepared and stored in the dark at around 20 °C and stirred until the trials. On the day of the trials, the stock solution was diluted in deionized water to 400 µg/L (feed solution, stirred and kept in the dark) and the diluted solution was then continuously added to the pilot feed tank with a peristaltic pump and mixed with the intake water.

### 2.2. Intake Water

Trials were conducted with two surface waters from Alcantarilha WTP (Águas do Algarve S.A.), herein designated as W1 and W2. As shown in Table 2, both waters presented low turbidity (<2 NTU), low-medium alkalinity (≤72 mg/L CaCO_3_) low NOM concentration (total organic carbon, TOC < 2 mgC/L) and low content of aromatic organic matter, the latter inferred by the low values of SUVA, the specific UV absorbance (given by absorbance at 254 nm/dissolved organic carbon, A254/DOC), below 2 L/(mg·m). W2 presents a somehow higher DOC content (+38%) and higher turbidity and A254 content (+167%), the latter parameter indicating a potentially higher pesticide-NOM competition for PAC adsorption, since PAC is known to preferentially adsorb A254-absorbing compounds [33,34].

### 2.3. PAC

PAC Norit SA Super (Cabot) was used, and its textural characterization was subcontracted to an external lab and performed by N_2_ adsorption-desorption isotherms at −196 °C in an automatic apparatus Micromeritics ASAP 2010 (Micromeritics, Norcross, GA, USA), as detailed in Viegas et al. [35]. PAC SA Super is alkaline at working pH (point of zero charge (pH_pzc_) of 11.3), presenting an average PAC particle of 15 μm, a surface area of 1126 m^2^/g and a total pore volume of 0.83 cm^3^/g. It has a high percentage of mesopores (mesoporous volume of 0.44 cm^3^/g and microporous volume of 0.39 cm^3^/g).

Two PAC options were tested: 10–12 mg/L inline PAC dosing into a loop before the membrane module, providing 1 min hydraulic retention time, or 8–10 mg/L PAC dosing to a stirred tank before the membrane, providing 2 h hydraulic retention time. A PAC slurry with 0.8 g/L was used, prepared with dechlorinated tap water.

### 2.4. Coagulant

A widely used metallic coagulant, alum, was used in PAC/coagulant/MF pilot at a 3 mg/L Al_2_O_3_ dose. Alum was only applied (inline dosing) in the runs with W2, hereafter referred as Alum/MF and PAC/Alum/MF.

### 2.5. PAC/(Alum)/MF Pilot

PAC/(Alum)/MF pilot is fully automated, remote controlled and with inline monitoring of pressure, flow rate, temperature, pH and turbidity. Pilot specific scheme may be found in [20]. The main component is a pressurized microfiltration module comprising three tubular MF (0.1 µm) ceramic (ZrO_2_/TiO_2_) membranes (1.2 m length and 25 mm diameter, KleanSep-Orelis) (Orelis Environment SAS, Salindres, France), with 19 channels each (3.5 mm diameter each), providing a total surface area of 0.75 m^2^. The membrane was operated in dead-end mode, at constant flux (133 L/(m^2^·h), in short lmh), with 60-min filtration cycles followed by backwash with 9.3 L permeate/m^2^ membrane area, at 1.4–1.5 bar (backwash time was varied to ensure this backwash specific volume). Chemically enhanced backwashing (CEB), either with sulphuric acid or sodium hypochlorite, was conducted after the trial end; it used approximately 22 L of permeate and comprised four steps: (1) flushing with water and cleaning agent; (2) 20–30 min. soaking with cleaning agent; (3) backwashing and (4) water permeation discarding the permeate. Based on the results of a long-term demonstration period conducted with similar waters [20], during W2 trial inline coagulation was conducted upstream the ceramic MF for membrane fouling control.

### 2.6. PAC/(Alum)/MF Trials

**Figure 1:**
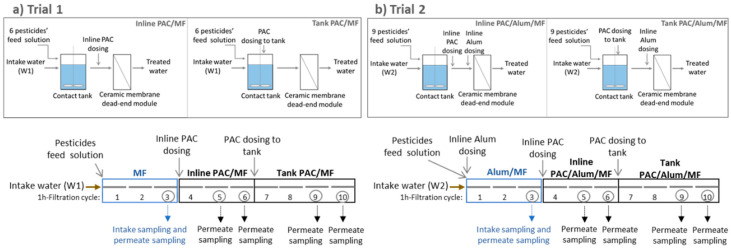
Scheme of PAC/(Alum)/MF process (top) and procedure followed (bottom) during trial 1 (**a**) and trial 2 (**b**) (10 × 1 h-filtration cycles, with 3 cycles without PAC, 3 cycles with inline PAC dosing and 4 cycles with PAC dosing to tank).

Two trials were conducted in PAC/(Alum)/MF pilot, with W1 or W2 intake water spiked with a mixture of 6 to 9 pesticides (single pesticide concentrations of 0.3–2.3 μg/L). Conditions used in trials 1 and 2 are summarized in Table 3 and schematically represented in Figure 1.

In trial 1, W1 was spiked with six pesticides (all compounds in Table 1 except chlortoluron, bentazone and dimethoate) until a 7.2 μg/L total-pesticide concentration. In trial 2, W2 was spiked with nine pesticides (all compounds in Table 1), until 10.3 μg/L total-pesticide concentration (Table 3). The spiking procedure was continuously ensured at pilot’s feed tank as follows: immediately before each trial, a pre-determined volume of the pesticides’ feed solution was supplemented to the feed tank to accelerate the desired steady state concentration of pesticides and allow short-term trials. Trials begun with the intake water (W1 in trial 1; W2 in trial 2) being continuously pumped to the feed tank and mixed with the pesticides’ feed solution continuously delivered by a peristatic pump (Figure 1). The pesticides-spiked water resulting from the pilot-scale trials was given an adequate destination previously approved by the local Environmental Agency. The terms agreed were that the waters produced in these trials were to be analysed for the spiked pesticides and, in case the concentrations were low as expected, the waters could be discharged provided low volumes were produced. Otherwise, these waters would have to be dealt as residues and given an adequate downstream treatment by a specialized external company. For this reason, waste minimization was a major factor and, as such, only short-term trials (around 10 h) were conducted.

Each trial comprised 10 cycles of 1 h-filtration each (Figure 1, bottom), starting with 3 cycles with no PAC addition (MF for W1; inline Alum/MF for W2), followed by 3 cycles with inline PAC dosing (inline PAC/MF for W1; inline PAC/Alum/MF for W2), and finally 4 cycles with PAC continuous dosing to the contact tank (tank PAC/MF for W1; tank PAC/Alum/MF for W2). Each filtration cycle was followed by a backwash to remove the accumulated solids and, at the end of trials, chemically enhanced backwashing (CEB) was performed. At the beginning of the first filtration cycle with PAC dosing to the contact tank, a pre-determined PAC mass was added to the tank for obtaining the desired steady-state PAC concentration. Afterwards, the PAC slurry was continuously dosed to the feed tank.

### 2.7. Sampling and Analysis

Composite samples from 5 portions (0.6 L–1.1 L each) gathered at 10 min, 20 min, 30 min, 40 min and 50 min of the filtration cycle were collected. To ensure stable conditions, sampling started after 1 or 2 stabilization (1h-filtration) cycles, depending on the systems’ hydraulic residence time (2 h considering the contact tank; 1 min in the inline dosing circuit). As indicated in Figure 1 (bottom), two stabilization cycles were conducted for (Alum)/MF, one for inline PAC/(Alum)/MF and two for tank PAC/(Alum)/MF. Therefore, feed samples were collected in the third filtration cycle; permeate samples were collected in the third filtration cycle (MF or Alum/MF, one sample), in the fifth and sixth filtration cycles (inline PAC/MF or inline PAC/Alum/MF, two samples) and in the last two filtration cycles (tank PAC/MF or tank PAC/Alum/MF, two samples) (Figure 1, bottom).

Samples were analysed for pesticides by ultra-high performance liquid chromatography coupled to tandem mass spectrometry (UPLC-MS-MS), subcontracted to an external laboratory certified for these parameters (Laboratório de Análises, IST, Lisbon). Pesticides were quantified on a triple quadrupole mass spectrometer (Waters ACQUITY TQD) combined with a Waters ACQUITY UPLC system (Waters, Milford, MA, USA) operating in multiple reaction monitoring (MRM) mode. A 20 µL sample was directly injected. The chromatographic separation was performed with an Acquity BEH C18 (75 mm × 2.1 mm i.d., 1.7 μm) column Waters Milford (MA, Ireland) set to 40 °C. The mobile phase components were (A) ultra-pure water + 0.1% formic acid + 0.05% ammonium and (B) acetonitrile + 0.1% formic acid. 8-min runs were conducted using 0.5 mL/min and the following elution gradient: 0 min, 95% A and 5% B; 5 min, 0% A and 100% B; 6 min, 95% A and 5% B; 8 min, 95% A and 5% B. The electrospray ionization (ESI) probe was operated in both negative (ESI-) and positive (ESI+) polarity modes. Instrument parameters were: capillary voltage at 0.5 kV; extractor voltage at 3.0 V; source temperature at 150 °C; desolvation temperature at 500 °C; the desolvation gas flow (nitrogen) at 1000 L/h and the cone gas flow at 50 L/h. Argon, used as the collision gas, was run at a collision gas pressure set at 3.5 × 10^−3^ mbar. Pesticides’ limit of quantification (LOQ) was 0.06 µg/L. The inter-day assay precision and accuracy were determined by analysing two different quality control (QC) samples over the years, LOQ (0.06 µg/L) and high QC (0.50 µg/L). The accuracy data were accepted if the accuracy values were within ± 20% deviation (80–120%) from the nominal concentrations, whereas the precision was measured as percentage standard deviation (%RSD) within ± 20%. Accuracy of 98–107% (at 0.06 µg/L pesticides) and 91–112% (at 0.5 µg/L) was verified for the targeted pesticides, while precision was 13–20% (at 0.06 µg/L) and 3–10% (at 0.5 µg/L). Other regular water quality parameters were analysed in Águas do Algarve accredited laboratory using standard methods for the examination of water and wastewater—SMEWW [36]. Turbidity was measured by nephelometry (ISO 7027-1:2016), TOC and DOC by high temperature combustion with infrared detector (EN 1484:1997), A254 by UV-VIS spectrophotometry (SMEWW 5910 B, (Thermo Fisher Scientific, Waltham, MA, USA), using quartz cells with 50 mm optical path length) and alkalinity by potentiometric titration (SMEWW 2320 B). DOC and A254 were measured on pre-filtered samples through 0.45-µm membrane filters.

### 2.8. Statistical Methods

The statistical significance (*p*-values) of differences in inlet and outlet concentrations of pesticides and in NOM and pesticides’ removal efficiencies in the three different configurations tested (MF vs. inline PAC/MF vs. tank PAC/MF or Alum/MF vs. inline PAC/Alum/MF vs. tank PAC/Alum/MF) was assessed through statistical tests using the Past 4.01 program. Briefly, one-way ANOVA (homogeneous variance with Levene’s test) or Welch F test (unequal variance) were conducted for normal distributions, and Kruskal Wallis test was used for not normal distributions (Shapiro-Wilk test, *p*-values < 0.05). Significance levels of 0.1 were applied instead of the usual 0.05 due to the low sample size [37].

## 3. Results and Discussion

### 3.1. Operational Results

**Figure 2:**
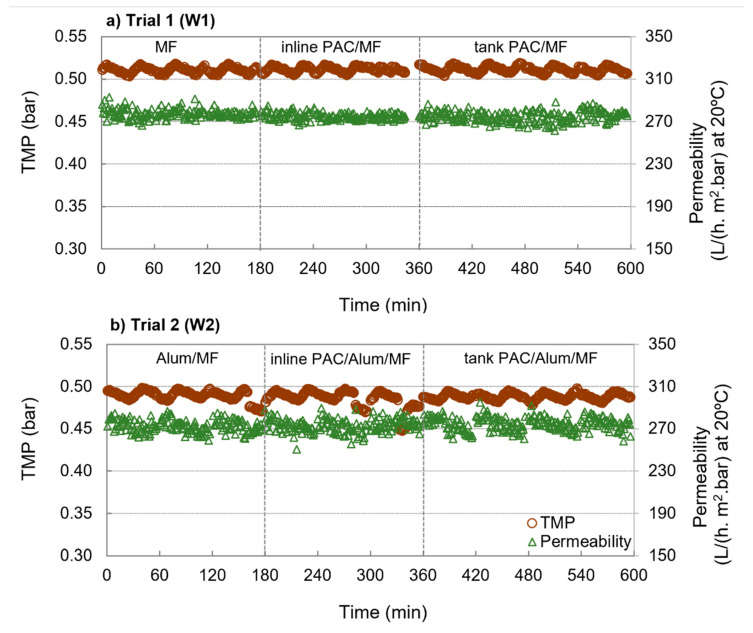
Transmembrane pressure (TMP) and membrane permeability during MF or PAC/MF filtration cycles with W1 (**a**) and during Alum/MF or PAC/Alum/MF filtration cycles with W2 (**b**); inline PAC dosing was tested in 3 filtration cycles and PAC dosing to contact tank in 4 filtration cycles.

The performances of MF and PAC/MF for W1 and Alum/MF and PAC/Alum/MF for W2 in terms of cycle-averaged inlet pressure, TMP, flux and specific flux (or permeability) at 20 °C is summarized in Table 4. Figure 2 depicts the transmembrane pressure (TMP) and permeability (at 20 °C) during MF or Alum/MF (3 cycles) and during PAC/MF or PAC/Alum/MF filtration cycles (7 cycles) of W1 and W2, the latter comprising both inline PAC dosing (3 cycles) and PAC dosing to contact tank (4 cycles).

Results showed similar transmembrane pressure and membrane permeability for MF, inline PAC/MF and tank PAC/MF with W1 and for Alum/MF, inline PAC/Alum/MF and tank PAC/Alum/MF with W2 (Figure 2 and Table 3), indicating no PAC-driven membrane fouling for both PAC dosing options (inline and tank). These results point out that, under the tested conditions, a porous cake layer (with no increased resistance and easily backwashed) was formed on top of the membrane surface either with continuous inline PAC dosing or with continuous PAC dosing to the contact tank. Although longer trials might be necessary to fully confirm it, the results are in agreement with our previous study with a PAC/FeCl_3_/ceramic MF pilot for treating a secondary effluent [24]. Moreover, our previous study with similar waters [20] showed that 6–24 mg/L PAC dosing to a contact tank did not promote membrane fouling and that treatment capacity, an indicator incorporating key aspects of process productivity and energy needs, kept constant or slightly increased with PAC dosing. In natural waters, contradicting results regarding PAC contribution to membrane fouling have been reported, likely due to complex interactions between NOM, metallic ions, PAC particles and membrane surfaces [10]. The present study tested a set of conditions derived from our previous work based on long-term operational data of PAC/(Alum)/ceramic MF [20] and taking into account the important aspects to minimize PAC cake fouling according to literature. Studies showed PAC addition may intensify membrane fouling in highly humic waters [38,39], yet to a lower extent for more hydrophilic membranes [39], and that the combination of metals and colloids accelerates PAC cake fouling, particularly when using large PAC particles (around 150 µm) [40]. The use of a hydrophilic membrane, the water characteristics, with low NOM content of low aromaticity, low Al, Fe and Mn ions’ concentrations (<10 µg/L Al, <0–21 µg/L Fe, <10 µg/L Mn), the PAC size (15 µm average diameter) and low PAC concentrations may therefore explain PAC cake fouling minimization in the present study. Our results are in agreement with other studies reporting very stable and high permeabilities when combining coagulation and PAC with ceramic MF [15,41,42].

### 3.2. Pesticides and Organic Matter Removal

**Figure 3:**
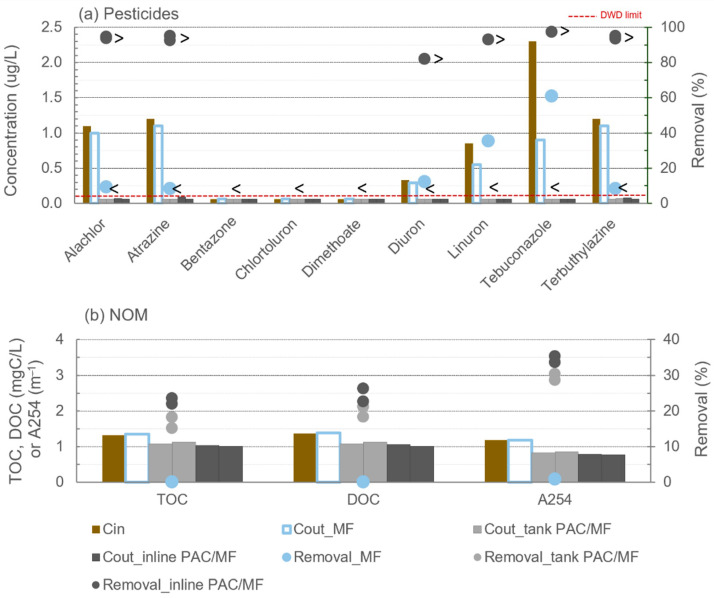
Pesticides’ concentrations (**a**) and NOM content (**b**) in trial 1: intake (W1) and permeate concentrations (bars) and removals (circles) after MF or PAC/MF with 10 mg/L PAC dosing, inline or to tank (“<” represents concentrations below LOQ and “>”removals computed with LOQ; doted red line is the Drinking Water Directive limit for pesticides).

**Figure 4:**
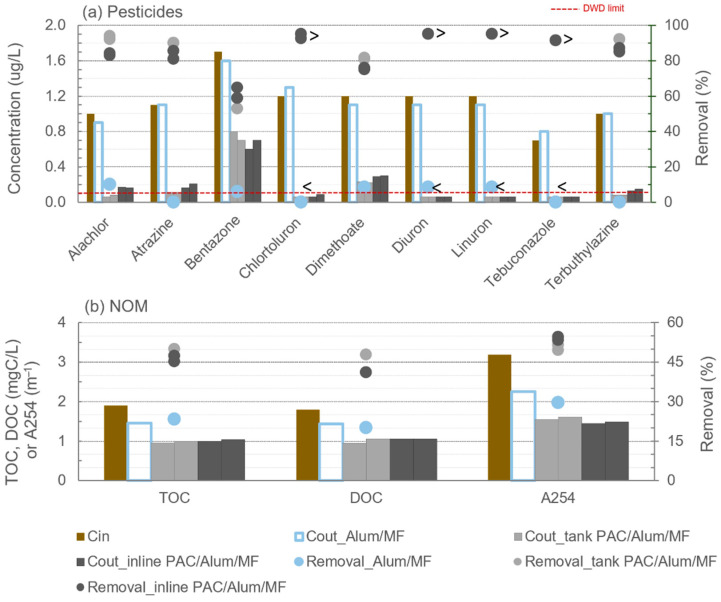
Pesticides’ concentrations (**a**) and NOM content (**b**) in trial 2: intake (W2) and concentrations (bars) and removals (circles) after Alum/MF or PAC/Alum/MF with 12 mg/L inline PAC dosing or 8 mg/L PAC dosing to tank (“<” represents concentrations below LOQ and “>”removals computed with LOQ; doted red line is the Drinking Water Directive limit for pesticides).

**Figure 5:**
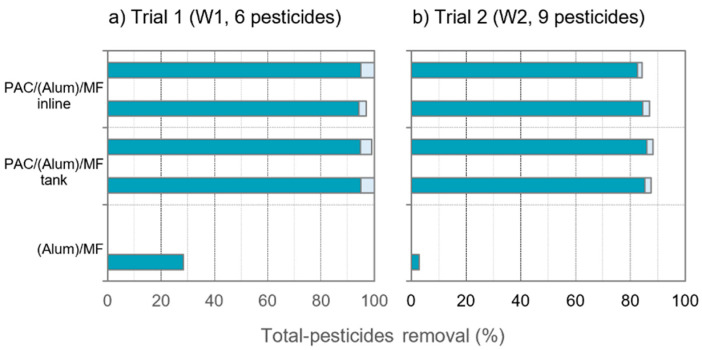
Total pesticides’ removal in trial 1, with W1 spiked with 6 pesticides (**a**) and in trial 2 with W2 spiked with 9 pesticides (**b**) (total-pesticides concentrations are given by summing the concentrations of all pesticides; due to some pesticides’ concentrations <LOQ in permeate, removals ranged between values computed with LOQ, represented in darker blue, and values computed with 0 µg/L, in light blue).

Concentrations of pesticides and NOM content (TOC, DOC and A254) in the intake, and concentrations in permeate and removals after MF and PAC/MF filtration cycles are shown in Figure 3 for trial 1 (with W1), comparing 10 mg/L PAC dosing either inline or to a contact tank. Similar representation for Alum/MF or PAC/Alum/MF filtration cycles is depicted in Figure 4 for trial 2 (with W2), with 8 mg/L PAC dosed to a contact tank and 12 mg/L inline PAC dosing. When the compound’s concentration was below the quantification limit (LOQ) the symbol “<” is represented. In these circumstances, the removal efficiency varied between the value computed with LOQ and the value computed with 0 µg/L which is reflected in Figure 5 relative to total-pesticides removal for trials 1 and 2. In Figure 3 and Figure 4 (for simpler graphics), the removal efficiency computed with LOQ was used together with the symbol “>”.

Our previous work studying PAC adsorption of pharmaceutical compounds and a similar pool of pesticides in the same concentration range, though with a different PAC and conventional application [11], showed no correlation between compounds’ removal efficiency and their initial concentration (C0) for all conditions tested. This was in agreement with several other studies concluding that, in natural waters and below a sufficiently low C0 value, the residual percentage concentration of microcontaminants is not a function of C0 for any activated carbon [11]. Therefore, even though different initial concentrations of pesticides were observed, this aspect mainly affects their outlet concentrations, and no relevant impact on removal efficiencies is expected for the concentration ranges studied.

Microfiltration, either alone or combined with coagulation, did not result in effective removal of pesticides (MF in Figure 3a; Alum/MF in Figure 4a). These results were expected as pesticides are too small to be retained by the 0.1 µm pore size membrane, and removal of microcontaminants by coagulation has been shown to be relevant only for very highly hydrophobic compounds (Log Kow > 6) and waters with high turbidity/NOM [7,43,44], which is not the case in the present study. With no PAC addition, removals below 10% were observed for all pesticides in the second trial (Alum/MF, Figure 4a), whereas a variation between 8% and 61% was observed in the first trial (MF, Figure 3a), the upper value for tebuconazole. The higher hydrophobicity (Log Kow = 3.7) and number of aromatic rings (2) of tebuconazole (Table 1) compared with the other target pesticides may justify some adsorption to NOM or to PAC residues not completely removed with the cleaning routines used in earlier experiments and still remaining in the pilot. Although with similar dominant NOM character, the relatively lower SUVA of W1 compared to W2 (0.9 L/(mg·m) vs. 1.8 L/(mg·m), Table 2) points to (a bit) less aromatic NOM in the first trial, making the pesticides-NOM hydrophobic interactions less likely and the second hypothesis (adsorption to PAC residues from earlier runs) as the most probable.

When 10 mg/L PAC was dosed to a 2h-contact tank or inline a major increase in pesticides’ removal from W1 was observed, without significant differences in pesticides’ removals (*p*-values ≥ 0.4) and outlet pesticides’ concentrations (*p*-value of 0.2 for those filtration cycles presenting variance) between the two PAC dosing options. All pesticides were very near or below LOQ with both tank PAC/MF and inline PAC/MF (Figure 3a), corresponding to removals ≥93% (for diuron the value represented is >82% due to the lower initial concentration). No NOM removal from W1 occurred with MF alone, but a significant increase (*p*-values < 0.007) was observed with both tank PAC/MF and inline PAC/MF, reaching 15–24% for TOC, 18–26% for DOC and 29–35% for A254 (Figure 3b). No significant differences of NOM removal were observed between the two PAC dosing modes (*p*-values > 0.4).

As previously referred, Alum was applied in trial 2, i.e., Alum/MF and PAC/Alum/MF were assessed for pesticides and NOM removal. Coagulant can influence pesticides’ removal by PAC/MF: (i) positively and directly, by adsorption of pesticides or pesticides-NOM complexes onto coagulation flocs, which may be retained by the MF membrane; (ii) positively and indirectly, by reducing NOM competition for PAC adsorption; (iii) negatively, by interfering with PAC adsorption.

On one hand, pesticides’ removal by coagulation depends on their adsorption onto colloids or NOM, a phenomenon apparently relevant only for very highly hydrophobic compounds (Log Kow > 6) and waters with high turbidity/high molar mass NOM [7,43,44], which are not the conditions studied. In fact, our previous work with PAC/coagulation/flocculation/sedimentation [11] corroborated coagulation inefficacy for removing the pesticides herein targeted from waters similar to W2. As so, in this study, pesticides’ removal by PAC/(Alum)/MF is considered to be mostly due to PAC addition, and Alum was mainly intended for membrane fouling control and was decided based on long-term pilot operational results [20]. Coagulant positive indirect effect on microcontaminants’ adsorption depends on water background and coagulation ability to remove the adsorptive NOM, frequently the smaller and more hydrophobic NOM fractions. Coagulation is more effective for removing high molar mass NOM [45,46], a fraction found irrelevant for reducing competitive adsorption [47] or associated with pore blockage [48], in this case NOM removal resulting in improved pesticide uptake. The low NOM content of the studied waters and the PAC/coagulant dosing sequence, i.e., coagulant addition after 2-h PAC contact (tank dosing) or coagulant and PAC simultaneous addition with only 1 min contact time prior to MF (inline), make us believe no significant coagulant effect occurred. Further testing is nevertheless necessary to validate this assumption.

On the other hand, coagulant may also negatively affect the removal of pesticides by interfering with PAC adsorption [49,50]), a more probable scenario for PAC and coagulant simultaneous addition (inline). Studies have shown conditions originating larger flocs to promote PAC entrapment into the floc structure and to reduce the mixing efficacy and the diffusion kinetics, an unlikely occurrence due to the low coagulant doses used and the low turbidity/low NOM water studied. Therefore, though no specific experiments were conducted in this study to assess coagulant influence onto pesticides’ removal by PAC/MF, overall, no substantial positive neither negative direct/indirect effect of coagulant is anticipated. Future research studies are recommended to confirm it.

In trial 2, a major increase in pesticides’ removal from W2 was also observed when 8 mg/L PAC was dosed to a 2-h contact tank, attaining ≥90% for all pesticides except dimethoate (81–82%) and bentazone (53–59%) (tank PAC/Alum/MF, Figure 4a). The much lower hydrophobicity of those two pesticides only added in trial 2 (Log Kow = 0.34 for dimethoate and Log Kow = 0.76 for bentazone, Table 1) and the inexistence of aromatic rings in dimethoate or the bentazone’s negative charge at neutral pH justify their lower adsorption onto PAC. Recent studies corroborate that pesticides with low Log Kow and low number of aromatic rings are more difficult to remove by PAC adsorption [7,11]. A similar behaviour was observed with 12 mg/L PAC inline dosing, attaining removals between 81% and >95% for all pesticides, except dimethoate (75–76%) and bentazone (59–65%) (inline PAC/Alum/MF, Figure 4a). Comparing inline vs. tank PAC dosing options (12 mg/L inline vs. 8 mg/L tank), removals apparently differed up to −12% for alachlor, −9% for atrazine, −7% for dimethoate, +12% for bentazone and −7% for terbuthylazine; however, statistical tests indicated no significant differences between the two dosing options in removals (*p*-values of 0.4–0.6, i.e., >0.1) nor in pesticides’ outlet concentrations (*p*-values ≥ 0.4) regardless of the 50% higher PAC dosed inline.

As depicted in Figure 4b, comparable NOM removals between tank and inline PAC dosing were also observed in trial 2 (*p*-values of 0.4–0.9), namely 47–50% (tank) vs. 45–47% (inline) for TOC, 41–48% (tank) vs. 41% (inline) for DOC and 50–51% (tank) vs. 53–54% (inline) for A254. Moreover, PAC addition, for both PAC dosing options tested, significantly (*p*-values < 0.08) enhanced the TOC, DOC and A254 removals also verified with Alum/MF (23% for TOC, 20% for DOC and 30% for A254). Studies have shown coagulation and PAC adsorption to complement each other in DOC removal, the former removing mostly high molar mass and the latter lower molar mass NOM [19,33].

Overall, and under the conditions tested, equivalent removals of pesticides and NOM were obtained with inline PAC dosing and to a contact tank in trial 1 using the same PAC dose, while in trial 2 a higher inline PAC dose was apparently required (50% higher in the conditions tested, though further testing with other PAC doses would be necessary to confirm it). Results from trial 1 are in agreement with those observed by Ivancev-Tumbas et al. [25] for p-nitrophenol removal from tap water, whereas results from trial 2 are more in agreement with our previous study [24], where PAC dosing to a contact tank yielded an added +15% to +18% pharmaceuticals’ removal from a secondary effluent. The tested pesticides are small, neutral (except bentazone) molecules, diffusing into the carbon pores faster than larger NOM molecules. The existence of a contact tank provides higher time for pesticides’ adsorption, but also provides an opportunity for the adsorption of NOM compounds with slower adsorption kinetics, promoting greater NOM adsorption and, potentially, higher NOM competition. Depending on the characteristics of NOM (e.g., size and aromaticity) and PAC (size, pore size distribution), longer contact times are sometimes advantageous for microcontaminants’ removal (particularly when they diffuse slowly) and sometimes not substantially useful.

In the European Union, the pesticides’ levels in water intended for human consumption are addressed by the Drinking Water Directive (DWD; [51]) and the Groundwater Directive (2006/118/EC). In both directives, concentrations of pesticides in drinking water may not exceed 0.1 µg/L (represented in Figure 3a and Figure 4a as a red line) for a single pesticide and 0.5 µg/L for total pesticides. Despite the high initial concentration of pesticides spiked in W1 (trial 1) and W2 (trial 2), 0.7–2.3 µg/L each pesticide, DWD limit was always complied with PAC/(Alum)/ceramic MF under the conditions tested in trial 1, but not for the conditions of trial 2, particularly for those compounds less amenable to adsorption (e.g., bentazone and dimethoate), indicating the need of higher PAC doses. The higher number of pesticides in trial 2 than in trial 1 (9 vs. 6), the higher total pesticides’ concentration (10.3 µg/L vs. 7.2 µg/L) and, particularly, the presence of pesticides less amenable to adsorption only in trial 2 (e.g., bentazone and dimethoate) partially justify differences between trials 1 and trial 2. Moreover, higher DOC and A254 loads were observed for PAC during trial 2(0.03–0.07 mgC/mg PAC in trial 2 (W2) vs. 0.03–0.04 mgC/mg PAC in trial 1 (W1); 0.06–0.09 m^−1^/mg PAC in trial 2 (W2) vs. 0.03–0.04 m^−1^/mg PAC in trial 1 (W1)), suggesting higher pesticide-NOM competition effects.

Summing up, very high removals of total-pesticides were verified in our study with PAC/(Alum)/ceramic MF (Figure 5), namely 95–100% (tank) and 94–100% (inline) in trial 1 or 85–88% (tank) and 83–87% (inline) in trial 2. Total-pesticides is given by summing the concentrations (conc.) of all targeted pesticides, and total pesticides removal is given by Equation (1):

(1) $$\mathrm{Total}\;\mathrm{pesticides}\;\mathrm{removal}\;\left(\%\right)=\frac{\mathrm{Total}\;\mathrm{pesticides}\;\mathrm{conc}.\;\mathrm{in}\;\mathrm{intake}\;-\;\mathrm{Total}\;\mathrm{pesticides}\;\mathrm{conc}.\;\mathrm{in}\;\mathrm{permeate}}{\mathrm{Total}\;\mathrm{pesticides}\;\mathrm{conc}.\;\mathrm{in}\;\mathrm{intake}}\times 100$$

Atrazine removals in our study (81–90%) were far higher than those reported by Humbert et al. [48] with 20–40 mg/L PAC dosing to a high DOC surface water (6 mgC/L) with 30 min contact (55–65%) and close to removals there obtained with 24 h contact time (85%). It is clear that even using a technology attaining high pesticides’ removal, such as PAC/(Alum)/MF, DWD limits may be difficult to comply in some circumstances, such as for waters with high concentrations of pesticides with low amenability to adsorption and/or with high NOM competition potential. Trial 2 is the example of the former, the compliance with DWD limits requiring higher PAC doses than those used herein.

Given the above results and considerations, a cost analysis was performed for three scenarios (Section 3.3), built based on the experimental results obtained in trials 1 and 2, combining the two water qualities tested, W1 (with low A254-absorbing NOM) and W2 (with a bit higher A254-absorbing NOM), and different types of pesticides, i.e., all pesticides amenable to adsorption or high loads of some compounds less amenable to adsorption (e.g., dimethoate and bentazone). As scenarios were built upon trial 1 and trial 2 results, further trials for confirming the assumptions should be conducted.

### 3.3. Cost Analysis

A cost analysis of PAC dosing was conducted for the full-scale operation of PAC/(Alum)/MF. The (Alum)/MF operational conditions considered were previously optimized during the pilot operational demonstration and are shown in Table 5. Further operational details can be found in [20].

The following assumptions were made for developing the cost functions:

- Technical assumptions
  – Plant lifespan (PLS): 40 years (typical values)
  – PAC slurry concentration: 30 g/L
  – PAC preparation residence time: 10 h
  – PAC preparation mixing gradient: 120 s^−1^
  – PAC contact time in the contact tank: 2 h
- Financial assumption
  – Finance rate (i): 1.87% (2017–2019 average finance rate of Águas de Portugal group)

The cost elements (capital expenditure costs, CAPEX, and operating expenditure costs, OPEX) considered for the PAC dosing are described in Table 6 and their derived cost functions in Equations (2)–(4).

(2) $$\mathrm{PAC}\;\mathrm{dosing}\;\mathrm{system}\;\mathrm{cost}\;\left(€\right)=\;25170\cdot \mathrm{PAC}\;\mathrm{dosing}\;\mathrm{rate}\;{\left(\mathrm{ton}/\mathrm{year}\right)}^{0.47}$$

(3) $$\mathrm{PAC}\;\mathrm{contact}\;\mathrm{tank}\;\mathrm{cost}\;\left(€\right)=\;3000\cdot \mathrm{contact}\;\mathrm{tank}\;\mathrm{volume}\;{\left(m^{3}\right)}^{0.59}$$

(4) $$\mathrm{Replacement}\;\mathrm{cost}\;\left(€/\mathrm{year}\right)=i\cdot \frac{{\left(1+i\right)}^{\mathrm{PLS}}}{{\left(1+i\right)}^{\mathrm{PLS}}-1}\cdot \left(\frac{\mathrm{PLS}}{\mathrm{CLS}}-1\right)\cdot \mathrm{asset}\;\mathrm{cost}\;\left(€\right)$$

Three scenarios were considered (Table 7), combining the two water qualities tested (W1 or W2), a high load of different pesticides (all amenable to adsorption or with high loads of some compounds less amenable to adsorption), and the two PAC dosing options:

- Scenario 1 considered an intake water similar to W1, with low A254-absorbing NOM content, and all pesticides amenable to adsorption, i.e., conditions analogous to trial 1. Equivalent removals of pesticides and NOM were obtained with inline PAC dosing and to a contact tank in trial 1 using the same PAC dose (10 mg/L); therefore, in this scenario, identical PAC dose was considered for both PAC dosing modes. As 8 mg/L was sufficient to comply with the DWD limit for the more amenable pesticides in trial 2, with more severe competing conditions than trial 1, this PAC dose was assumed adequate for scenario 1.
- Scenario 2 considered an intake water similar to W2, with a bit higher content of A254-absorbing NOM, and containing a high load of pesticides amenable to adsorption, i.e., conditions analogous to trial 2 but disregarding the pesticides less amenable to adsorption (dimethoate and bentazone). Results of trial 2 showed the compounds amenable to adsorption to be highly removed (below the DWD limits); thus, those PAC dosing conditions were considered, i.e., 12 mg/L inline and 8 mg/L to tank.
- Scenario 3 considered an intake water similar to W2, with a bit higher A254-absorbing NOM content, and containing a high pesticides’ load, with some compounds less amenable to adsorption, i.e., conditions analogous to trial 2. Results of trial 2 showed that 50% higher inline PAC dose than tank dose (12 mg/L inline vs. 8 mg/L to tank) reached identical removals of pesticides and NOM. As the DWD limits were not complied for the less amenable compounds in trial 2, 50% higher PAC doses than those used in trial 2 were assumed in scenario 3, i.e., 18 mg/L (inline) and 12 mg/L (tank).

**Figure 6:**
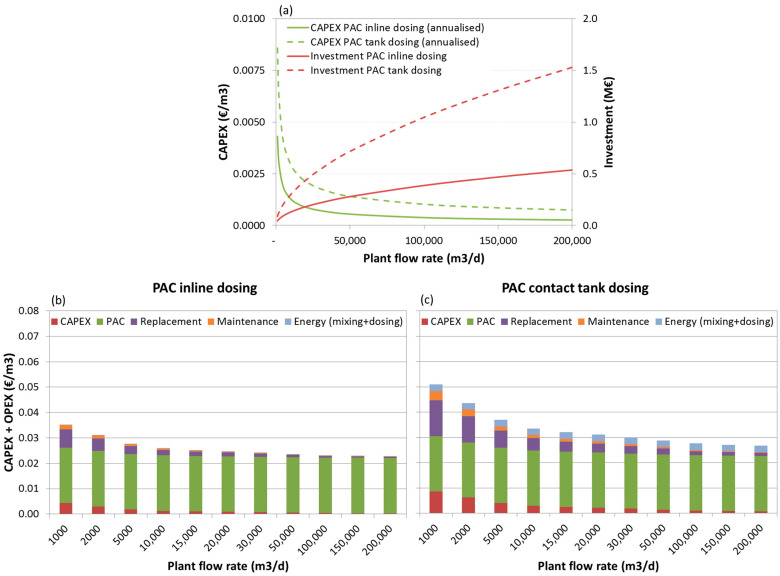
Investment costs and annualised CAPEX (**a**) and total (CAPEX+OPEX) production costs breakdown for scenario 1, dosing 8 mg/L PAC inline (**b**) and 8 mg/L PAC to tank (**c**).

**Figure 7:**
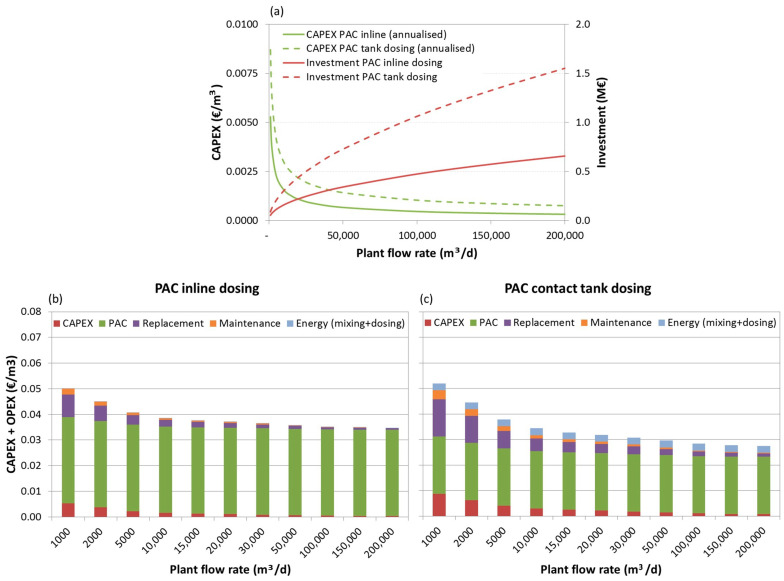
Investment costs and annualised CAPEX (**a**) and total (CAPEX+OPEX) production costs breakdown for scenario 2, dosing 12 mg/L PAC inline (**b**) and 8 mg/L PAC to tank (**c**).

**Figure 8:**
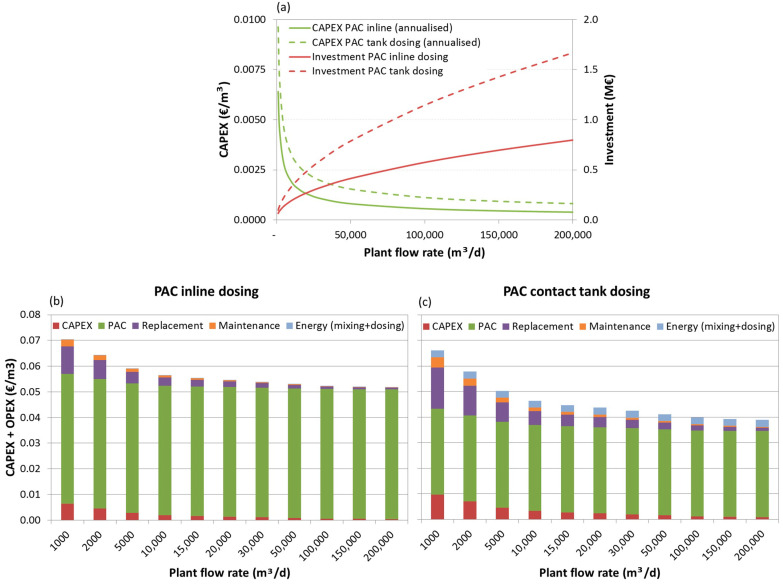
Investment costs and annualised CAPEX (**a**) and total (CAPEX+OPEX) production costs breakdown for scenario 3, dosing 18 mg/L PAC inline (**b**) and 12 mg/L PAC to tank (**c**).

The investment costs, the annualized CAPEX and the total (CAPEX+OPEX) production costs breakdown for the three scenarios are depicted below, as functions of the plant flow rate, in Figure 6, Figure 7 and Figure 8, respectively.

Analysing the investment needs (Figure 6a, Figure 7a, Figure 8a), it can be seen that dosing PAC to a contact tank increases the investment by 50%, for lower capacity plants in scenarios 2 and 3, and to 200%, for higher capacity plants in scenario 1. Furthermore, for this dosing option the weight of capital related costs (annualised CAPEX, replacement costs and maintenance) may increase considerably, especially for very low capacity plants (<2000 m^3^/d) with low PAC dosing needs.

Concerning the costs breakdown (Figure 6b,c, Figure 7b,c, Figure 8b,c), regardless of the plant size and scenario, the higher share is the PAC acquisition cost, representing 60% (for small scale plants) to 98% (for large scale plants) of the costs when inline PAC dosing is used, and 43% (for small scale plants) to 87% (for large scale plants) of the costs when PAC is dosed to a contact tank.

The total PAC dosing costs (CAPEX and OPEX) are estimated in 0.02–0.04 €/m^3^ (depending on the plant size, from large scale to small-scale plants, respectively) for scenario 1, 0.03–0.05 €/m^3^ for scenario 2 and 0.04–0.07 €/m^3^ for scenario 3, thus much depending on the nature of the pesticides (more or less amenable to adsorption) and on the A254-absorbing NOM.

When comparing PAC dosing options, it can be concluded that when PAC dose is the same in both options (as in scenario 1) or the required doses are low, though 50% higher in inline dosing (scenario 2), and the plant is small, inline PAC dosing would be more cost-effective. However, for larger plants under scenario 2 or if PAC doses are higher for both options and particularly for inline dosing, as in scenario 3, PAC dosing to a contact tank will be more cost effective.

## 4. Conclusions

Two pilot trials of PAC/(Alum)/ceramic MF were conducted with two surface waters (W1 and W2) spiked with a mixture of pesticides to compare two PAC dosing modes: inline and to a contact tank. The short-term trials conducted with these low turbidity/low NOM waters showed no PAC-driven membrane fouling for both PAC dosing options.

Differences of pesticides and NOM removal between inline PAC dosing and PAC dosing to contact tank depended on pesticides’ amenability to adsorption and NOM characteristics (e.g., A254). In trial 1, with low A254-absorbing NOM and all pesticides amenable to adsorption, high removals of total-pesticides were reached, 95–100% (tank) and 94–100% (inline), with no significant differences in pesticides and NOM removal between the two PAC dosing options. In trial 2, with a bit higher NOM competition (>DOC; >>A254) and high concentration of some pesticides less amenable to adsorption, total-pesticides’ removals somewhat decreased, 85–88% (tank) and 83–87% (inline), with a higher inline PAC dose apparently being required to match pesticides’ and NOM removal with tank dosing. Pesticides with low hydrophobicity (low Log Kow) and low number of aromatic rings or negative charge presented the poorest removals.

A considerable NOM removal was observed with the hybrid processes of PAC/Alum/MF or PAC/MF, 41–48% DOC and 50–54% A254 for the former, and 18–26% DOC and 29–35% A254 for the latter, which might be relevant for minimizing oxidation by-products after final disinfection and for water stability during distribution. Moreover, PAC complemented coagulation, adding 20–28% NOM removal.

The cost analysis undertaken showed that, for controlling high loads of pesticides, total PAC dosing costs (CAPEX and OPEX) range from 0.02 €/m^3^ to 0.04 €/m^3^ for large-scale plants and from 0.03 €/m^3^ to 0.07 €/m^3^ for small-scale plants, the lower end of the intervals corresponding to waters containing low A254-absorbing NOM and only pesticides amenable to adsorption and the higher end of the intervals to waters containing higher A254-absorbing NOM and pesticides less amenable to adsorption. For the first scenario, PAC inline dosing shows to be more cost-effective, whereas PAC dosing to a contact tank proves to be advantageous for the latter.

To comply with the Drinking Water Directive limits for pesticides, the control of those less amenable to PAC adsorption or in waters with high NOM competition may require higher PAC doses than those herein tested. Moving towards mitigating pollution at source and regular monitoring are therefore extremely important measures and a multi-barrier treatment approach might be advisable if highly challenging waters/conditions are expected to be found in drinking water production.

Further research comparing tank and inline PAC dosing in pressurized adsorption/membrane hybrid processes, at pilot scale and with natural waters, is recommended to test: (i) different PAC doses and confirm the effect above proposed; (ii) different water matrixes; (iii) the removal of other microcontaminants with different characteristics and amenabilities to PAC adsorption; iv) the removal of other key-parameters of water quality (e.g., NOM, trihalomethane formation potential, viruses). Additional tests are also recommended to confirm the coagulant influence on pesticides removal by PAC/MF and its dependence on water quality.

## Figures and Tables

**Table 1:** Pesticides used to spike intake waters feeding PAC/coagulation/ceramic MF pilot.

Compound	Molar Mass (Da)	Log Kow	Log D (at pH 7.8)	Charge (at pH 7.8)	Aromatic Rings
Alachlor	270	3.59	3.59	0	1
Atrazine	216	2.20	2.20	0	1
Bentazone	240	0.76	−0.19	−1	1
Chlortoluron	213	2.44	2.44	0	1
Dimethoate	229	0.34	0.34	0	0
Diuron	233	2.53	2.53	0	1
Linuron	249	2.30	2.30	0	1
Tebuconazole	308	3.69	3.69	0	2
Terbuthylazine	230	2.48	2.48	0	1

Retrieved from Chemspider database (experimental values or ACD/labs predictions, when experimental values not available).

**Table 2:** Water characteristics fed to PAC/coagulation/MF pilot during trials.

Water	°C	pH	Alkalinity (mg CaCO_3_/L)	Turbidity (NTU)	TOC (mg C/L)	DOC (mg C/L)	A254 (m^−1^)	SUVA (L/(mg·m))
W1	16	7.4	72	<0.1	1.4	1.3	1.2	0.9
W2	17	7.5	60	1.6	1.9	1.8	3.2	1.8

**Table 3:** Summary of the two trials conducted in PAC/(Alum)/MF pilot.

Trial	Intake Water	Pesticides Spiked to Intake Water	Total-Pesticides (µg/L)	Filtration Cycles	Inline PAC Dosing (mg/L PAC)	Tank PAC Dosing (mg/L PAC)	Inline Alum Dosing (mg/L Al_2_O_3_)
1	W1	6 pesticides (all those listed in Table 1 except chlortoluron, bentazone and dimethoate)	7.2	MF: 3 cycles Inline PAC/MF: 3 cycles Tank PAC/MF: 4 cycles	10	10	0
2	W2	9 pesticides (all those listed in Table 1)	10.3	Alum/MF: 3 cycles Inline PAC/Alum/MF: 3 cycles Tank PAC/Alum/MF: 4 cycles	12	8	3

**Table 4:** Performances of (Alum)/MF and PAC/(Alum)/MF with tank and inline PAC dosing configurations (AVG—average values, SD—standard deviation and n—number of measurements).

Parameter	Trial 1 (W1)						Water 2 (W2)					
	MF		Inline PAC/MF		Tank PAC/MF		Alum/MF		Inline PAC/Alum/MF		Tank PAC/Alum/MF	
	AVG	SD (n = 166)	AVG	SD (n = 158)	AVG	SD (n = 227)	AVG	SD (n = 168)	AVG	SD (n = 167)	AVG	SD (n = 228)
Inlet pressure (bar)	0.51	0.03	0.51	0.01	0.53	0.01	0.51	0.00	0.51	0.00	0.51	0.00
TMP (bar)	0.51	0.00	0.51	0.00	0.51	0.00	0.49	0.00	0.49	0.01	0.49	0.00
Flux (lmh)	142	2	141	2	141	2	133	1	133	1	134	0
Specific flux (lmh/bar)	278	4	276	3	276	5	273	0	273	2	275	0

**Table 5:** Optimized (Alum)/MF operational conditions [20] considered for the cost analysis.

Water	Flux (at 20 °C) (L/(m^2^·h))	TMP (bar)	Filtration Cycles Duration (h)	CEB (no./d)	Alum (mg/L)
W1	283	0.55	3	0.7	-
W2	174	0.77	2	1	0.82

**Table 6:** Cost elements considered for the PAC dosing.

Cost Element	Individual Elements & Quantification	Cost
**CAPEX** PAC dosing system	Comprising equipment (2 positive displacement pumps (efficiency 80%), silo, basin, pipes and valves), instrumentation and control and a building, 15 years lifespan (LS)	Cost function derived from PT installation costs (Equation (2), adapted from [20])
**CAPEX** PAC contact tank	Stainless steel stirred tank reactor, 15 years lifespan	Cost function expressed by Equation (3) [20]
**OPEX** PAC acquisition	Cabot Norit SA Super	2.44 €/kg (cost provided by PT supplier)
**OPEX** replacement of assets	Annualised costs of replacement of equipment	Cost function considering asset cost, plant and components lifespan (CLS), finance rate (Equation (3))
**OPEX** energy for PAC mixing & pumping	Energy consumption for PAC mixing, in the slurry preparation basin (inline dosing) and in the contact tank (tank dosing), and pumping to the MF system	0.08 €/kWh (cost provided by the water utility)
**OPEX** maintenance costs	Function of CAPEX	1.5% of the total capital costs/year

**Table 7:** Three scenarios considered for cost analysis.

Scenario	Water	Pesticides	PAC Dosed (mg/L)	
			Inline	to a 2h-Contact Tank
1	Analogous to W1	High pesticides’ load, all compounds amenable to adsorption	8	8
2	Analogous to W2	High pesticides’ load, all compounds amenable to adsorption	12	8
3	Analogous to W2	High pesticides’ load, with some compounds less amenable to adsorption	18	12

## Data Availability

Data is contained within the article.
